# Effects of transcranial magnetic stimulation on anhedonia in treatment resistant major depressive disorder

**DOI:** 10.1002/brb3.2329

**Published:** 2021-08-28

**Authors:** Andrew M. Fukuda, Jee Won Diane Kang, Asi Polly Gobin, Eric Tirrell, Fatih Kokdere, Linda L. Carpenter

**Affiliations:** ^1^ Butler Hospital TMS Clinic and Neuromodulation Research Facility Providence Rhode Island USA; ^2^ Department of Psychiatry and Human Behavior Alpert Medical School of Brown University Providence Rhode Island USA

**Keywords:** anhedonia, depression, naturalistic, transcranial magnetic stimulation

## Abstract

**Background:**

Anhedonia is one of the defining features of depression but it remains difficult to target and treat. Transcranial magnetic stimulation (TMS) is a proven treatment for depression, but its effects on anhedonia and whether anhedonia can be used as a predictive biomarker of response is not well known.

**Methods:**

Snaith–Hamilton Pleasure Scale was administered to patients with depression before and after a standard course of TMS in a naturalistic outpatient setting.

**Results:**

144 patients were analyzed. There was an overall significant improvement in anhedonia from pre‐ to post‐treatment (7.69 ± 3.88 vs. 2.96 ± 3.45; *p* < .001). Significant correlations between improvements in anhedonia and other depressive symptoms were present (*r* = 0.55, *p* < .001). Logistic regression revealed that baseline anhedonia severity was not a significant predictor of clinical outcome.

**Conclusion:**

This is the first large, naturalistic study examining the effects of standard, non‐research TMS on anhedonia. Among depressed patients, TMS resulted in significant improvements in anhedonia. Patients with severe baseline anhedonia had an equal chance of achieving clinical response/remission. Patients with anhedonia should not be excluded from treatment if they are safe for outpatient care and otherwise appropriate candidates for treatment.

## Graphical abstract

Here, we report our findings on the decrease in anhedonia symptoms in patients with treatment resistant depression receiving repetitive transcranial magnetic stimulation (TMS) in a naturalistic outpatient setting. The anhedonia symptoms were measured using a well validated self‐report tool specific for anhedonia: the Snaith–Hamilton Pleasure Scale. Furthermore, we report that baseline anhedonia symptoms and severity were not predictive of clinical outcome of TMS and those with severe anhedonia were just as likely to achieve clinical response/remission.

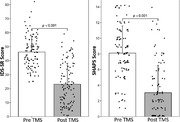

## 1 INTRODUCTION

Depression affects over 320 million people globally and is the leading cause of morbidity worldwide (Depression & Other Common Mental Disorders: Global Health Estimates, 2017). Approximately 30% of patients reach clinically significant remission after an adequate trial of antidepressant medication (Trivedi et al., 2006). Alongside pharmacotherapy and psychotherapy, neuromodulatory treatments such as electroconvulsive therapy (ECT) and transcranial magnetic stimulation (TMS) have become part of the standard of care for depression. TMS is a non‐invasive approach to brain stimulation that uses pulsed magnetic fields applied over the scalp to induce current in targeted areas of the cortex, such as the dorsolateral prefrontal cortex (DLPFC) for major depressive disorder (MDD) (McClintock et al., 2018). While the efficacy of TMS for MDD has been established in large, multi‐site randomized clinical trials and naturalistic studies (Carpenter et al., 2012; Dunner et al., 2014; George et al., 2010; O'Reardon et al., 2007), questions still remain about where it fits in an overall MDD treatment algorithm, particularly when treating patients with severe “melancholic” depression with marked anhedonia, that is, patients who have historically been considered among the most appropriate candidates for ECT.

Anhedonia, one of the hallmark symptoms of depression, is defined as “markedly diminished interest or pleasure in all, or almost all, activities most of the day, nearly every day” in the Diagnostic and Statistical Manual of Axis I Disorders, 5th Edition (American Psychiatric Association, 2013). In pharmacotherapy trials, anhedonia has been shown to be a predictor of inferior outcomes (McMakin et al., 2012; Uher et al., 2012). Anhedonia may be resistant to improvement even when other symptom domains of MDD improve with treatment (Cao et al., 2019), and anhedonia is associated with poor psychosocial functioning, hopelessness, and suicide completion (Bonanni et al., 2019; Buckner et al., 2008). While anhedonia is a symptom construct of considerable interest across a range of psychopathologies and diagnoses (Lambert et al., 2018), a comprehensive literature review of anhedonia symptomatology in neuromodulatory treatments in 2019 found only seven papers comprising 201 subjects, of which only 58 had a diagnosis of treatment resistant depression. (Spano et al., 2019).

Compared to studies of anhedonia in the context of pharmacotherapy, relatively few have examined the effect of TMS therapy on anhedonia (Spano et al., 2019). Several studies to date report on the utility of anhedonia as a predictor of TMS treatment outcome, but these studies defined anhedonia by selecting several items from a general measure of depression severity rather than with anhedonia‐specific measures (Downar et al., 2014; Krepel et al., 2020; Rostami et al., 2017). Using a subscale of the Beck Depression Inventory (BDI), Downar et al. (2014) found that more anhedonia symptoms and decreased reward circuit connectivity were associated with nonresponse when TMS was applied to the dorsomedial prefrontal cortex (DMPFC), but their course of treatment included only 20 sessions. Based on regulatory clinical trials and FDA labeling of commercial TMS devices, most American TMS clinicians do not routinely stimulate that brain region (DMPFC), and 30 or more sessions are delivered in a typical acute course in the United States. Since prediction of nonresponse differs as a function of the total number of treatment sessions (Beck et al., 2019), data are still needed to determine whether patients with marked anhedonia are likely to benefit from a standard course of TMS therapy for MDD delivered to the most commonly used target (DLPFC).

We analyzed data from a large naturalistically treated sample assessed with a validated anhedonia scale to evaluate whether a standard course of TMS to the left DLPFC improves anhedonia in MDD patients, and to test whether baseline anhedonia severity can be used as a predictive biomarker for TMS treatment outcomes.

## 2 METHODS

Data were retrospectively extracted from medical records for 144 naturalistically treated adult outpatients in the Butler Hospital TMS Clinic receiving TMS for the first time and who completed a self‐report anhedonia scale as part of their clinical assessment battery. The anhedonia scales were offered as part of our clinic's standard assessment battery from August 2016 to April 2020 to patients who were receiving TMS treatment. All met insurance criteria for TMS coverage, that is, primary diagnosis of MDD, resistance to at least one adequate antidepressant medication trial or a documented history of intolerance to antidepressant medications, and absence of psychotic features. Those with significant comorbid neurological disorders, such as seizure disorder or intracranial pathology were excluded. Diagnosis was made by clinical interview with a psychiatrist specializing in mood disorders, with collateral supporting information provided by past treatment records and referring clinicians. All patients were on stable but ineffective medication regimens at time of referral to the clinic and were directed not to change medications during the course of TMS, per standard clinical practice.

### 2.1 TMS protocol

All patients were treated with a figure‐8 coil over the left DLPFC at an intensity of 120% relative to resting motor threshold. Following motor threshold determination, the series was initiated with a standard 10 Hz protocol and a total of 3000–4000 pulses were delivered per session. In some cases where 10 Hz was poorly tolerated, patients were switched to 5 Hz stimulation, per our clinic's standard protocol (Philip et al., 2016), targeting the same stimulation site and with the same total number of pulses per session. Treatments were scheduled for five sessions/week, typically for six weeks, followed by an additional six sessions over 3 weeks of tapering frequency. Patients who reached remission before session #30 were offered early transition to the taper phase.

### 2.2 Clinical assessment

Depression severity was measured via the Inventory of Depressive Symptomatology‐Self Report (IDS‐SR) (Rush et al., 1996) scale at baseline (prior to first TMS) and after the final session in the taper phase. Clinical response was defined as ≥50% reduction in score from baseline to post‐treatment, and remission was defined by post‐treatment IDS‐SR score ≤14 (Rush et al., 1996). The IDS‐SR is a 28‐item measure which contains four items that assess hedonic drive. The four anhedonic items on the IDS‐SR were determined to be items 8 (Response of Your Mood to Good or Desired Events), 19 (General Interest), 21 (Capacity for Pleasure or Enjoyment, excluding sex), and 22 (Interest in Sex). We also calculated a revised total score (IDS‐SR_24_) which excludes those four items and sums the remaining 24 items to use when comparing the severity of anhedonia to the severity of other depressive symptoms.

At baseline and post‐treatment, patients also routinely completed the Snaith–Hamilton Pleasure Scale (SHAPS), a widely used 14‐item self‐report scale measuring the consummatory pleasure aspect of anhedonia (Snaith et al., 1995). Each item is a statement to which the patient responds with one of four response categories: “Definitely Agree,” “Agree,” “Disagree,” and “Strongly Disagree.” To derive a total SHAPS score, items with responses of “Disagree” or “Strongly Disagree” are recoded as having a value of zero, and items with “Agree” or “Definitely Agree” are recoded as having a value of 1, then the 14 items are summed. The SHAPS total thus ranges from 0 to 14, with higher scores reflecting higher levels of current anhedonia. Based on previously published psychometric studies (Nakonezny et al., 2010; Nakonezny et al., 2015), a total SHAPS score of 2 or less indicated a non‐anhedonic state, and a score of 6 or greater defined severe anhedonia, while scores between 3 and 5 were reflective of a mild–moderate degree of anhedonia.

### 2.3 Statistical analysis

Categorical (responders, remitters, SHAPS severity) and continuous (baseline‐to‐endpoint %changefor IDS‐SR_28_ and SHAPS total scores) outcomes for the entire sample were examined with simple descriptive statistics. When applicable, results are reported as mean ± standard deviation. Paired *t*‐tests evaluated baseline‐to‐endpoint change in mean values. Cohen's *d* was calculated to determine effect sizes. Statistical significance was defined at 0.05 and two tailed. Pearson correlation tests were performed to evaluate associations between SHAPS and IDS‐SR scores as well as the relationship between the two scales with regard to %change from baseline to treatment endpoint. To further examine changes in anhedonia compared to other depressive symptoms, the same correlations were also performed between SHAPS and IDS‐SR scores with the four anhedonic items removed (IDS‐SR_24_).

To evaluate whether level of anhedonia was associated with different clinical outcomes, we examined categorical outcomes (response, remission) for three patient subgroups defined by anhedonia severity (none, mild/moderate, or severe) with Chi‐square analysis. Then, to determine whether baseline anhedonia score was predictive of TMS treatment outcomes, logistic regression was performed with clinical response (defined by IDS‐SR_28_) as the dependent variable, and baseline anhedonia score (0 to 14), sex (male or female), and age (years) as covariates. The same regression model was run with remission as the dependent variable. To further examine the relationship of these predictors with depression symptoms outside of anhedonia, the same model was also performed with clinical response using the IDS‐SR with anhedonic items removed (IDS‐SR_24_) as the dependent variable. All data analysis was performed using SPSS statistical software (SPSS Inc. Chicago, IL).

## 3 RESULTS

### 3.1 Demographic and clinical outcome

**FIGURE 1:**
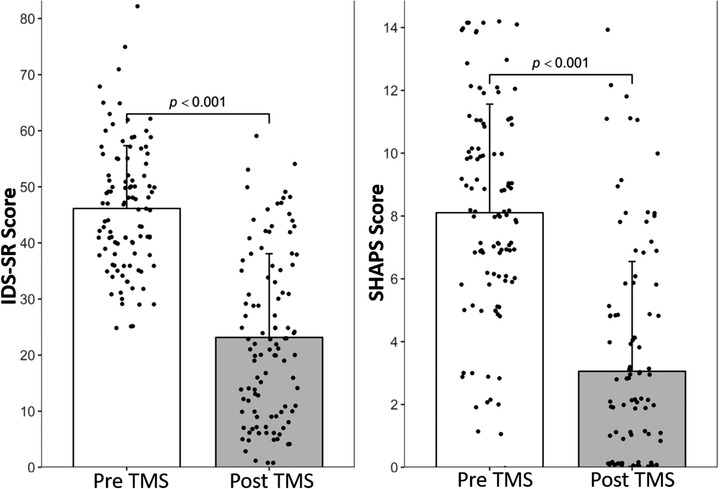
Improvement in overall depressive symptom scores and anhedonia scores. (a) There was a significant improvement in depressive symptoms as seen via a decrease in IDS‐SR scores from pre‐TMS (46.36 ± 11.19) to post‐TMS (25.40 ± 16.00), (*p* < .001, Cohen's *d* = 1.49). (b) A significant improvement in anhedonia symptoms were also observed via a decrease in SHAPS scores from pre‐TMS (8.10 ± 3.46) to post‐TMS (3.06 ± 3.49), (*p* < .001, Cohen's *d* = 1.45). Error bars are standard deviations and each point represents individual scores. IDS‐SR, Inventory of Depressive Symptomatology‐Self Report; SHAPS, Snaith–Hamilton Pleasure Scale; TMS, transcranial magnetic stimulation

144 patients who received their first TMS treatment series between August 2016 and April 2020 completed a baseline IDS‐SR and SHAPS; a subset of these (*n* = 105) also completed a post‐treatment SHAPS and IDS‐SR. Average age at start of treatment was 43.86 ± 16.42 years; 68.06% (*n* = 98) were female; 22.22% (*n* = 32) had at least one past ECT treatment; and 62.50% (*n* = 90) had at least one prior inpatient psychiatric hospitalization. The average number of TMS treatments was 35.49 ± 6.65 treatments. 98 (68.06%) patients received exclusively or predominantly 10 Hz stimulation, and 46 (31.94%) received predominantly 5 Hz stimulation. There was a significant decrease from baseline IDS‐SR score (46.36 ± 11.19) to final IDS‐SR score (25.40 ± 16.00) (*t *= 15.89, *p* < .001, Cohen's *d* = 1.49) (Figure 1), reflecting a mean 45.25% ± 31.89% decrease following TMS for the full sample. Overall, 45.83% (*n *= 66) achieved response as defined by the IDS‐SR and 31.94% (*n* = 46) met criteria for remission of their depressive episode following TMS.

### 3.2 Effect of TMS on anhedonia

**FIGURE 2:**
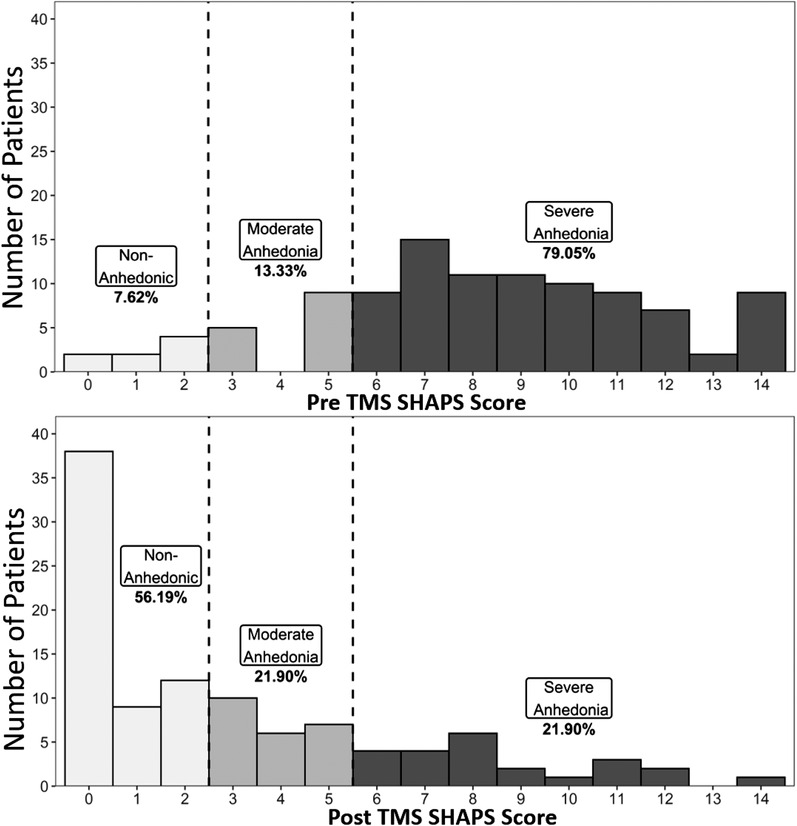
Changes in distribution of anhedonia severity with transcranial magnetic stimulation. Shown is the distribution pattern of anhedonia severity using pre‐determined anhedonia severity categories using SHAPS score cut‐offs as visualized from (a) pre‐TMS and (b) post‐TMS. SHAPS, Snaith–Hamilton Pleasure Scale; TMS, transcranial magnetic stimulation

Among the 105 patients who had both pre‐ and post‐treatment data, mean SHAPS was 8.10 ± 3.46 at baseline, and post‐treatment mean SHAPS was 3.06 ± 3.49, representing a significant reduction in anhedonia over time (*t* = 12.90, *p* < .001, Cohen's *d *= 1.45) (Figure 1). Baseline‐to‐post TMS mean %change on SHAPS was 58.55% ± 48.32%. At baseline only 8 patients (7.62 %) in the sample were classified by SHAPS as “non‐anhedonic” while 14 patients (13.33%) had “mild/moderate anhedonia,” and 83 patients (79.05%) had “severe anhedonia.” After treatment, 59 patients (56.19%) were classified as “non‐anhedonic,” 23 patients (21.90%) had "mild/moderate anhedonia,” and 23 patients (21.90%) were “severely anhedonic” (Figure 2).

### 3.3 Correlation between SHAPS and IDS‐SR improvement

There was a statistically significant positive correlation between SHAPS and IDS‐SR scores after TMS (*r* = 0.67, *p* < .001) and percent improvement over time (*r* = 0.59, *p* < .001), signifying that the degree of improvement with TMS seen in overall depressive symptoms measured via IDS‐SR and anhedonia as measured via SHAPS was similar.

**FIGURE 3:**
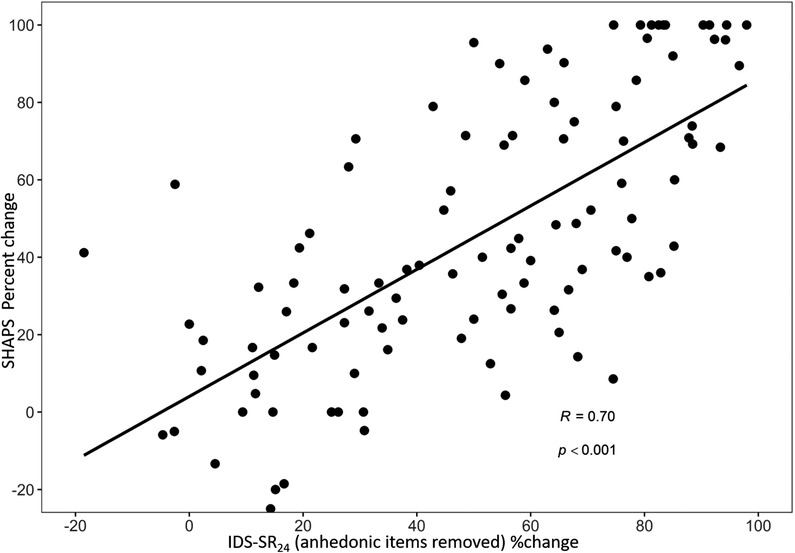
Correlation between changes in anhedonia symptom and the rest of the depressive symptoms. A significant correlation was present between percent change in SHAPS score and IDS‐SR_24_ (IDS‐SR without the anhedonic items) with TMS. IDS‐SR_24_: inventory of Depressive Symptomatology‐Self Report without the four anhedonic items; SHAPS: Snaith–Hamilton pleasure scale, , TMS, transcranial magnetic stimulation

In order to tease out the possible redundancy in measuring the anhedonic items in the IDS‐SR, which may inflate a significant correlation between SHAPS and IDS‐SR, we also performed a correlation between the scales with the four anhedonic items removed from the IDS‐SR. When responses to the four IDS‐SR items assessing hedonic drive were subtracted from the overall IDS‐SR scale total, significant correlations were still present between IDS‐SR_24_ scores and SHAPS scores at post‐TMS (*r* = 0.63, *p* < .001), and in overall %change over time (*r* = 0.56, *p* < .001) (Figure 3). Thus, the degree of improvement in anhedonia was comparable to the degree of improvement in non‐anhedonic depressive symptoms as well.

### 3.4 Predictive utility of baseline anhedonia on clinical outcome

The likelihood of achieving response with TMS did not differ based on baseline anhedonia severity category assessed via a 3 × 2 Chi‐square; 52.94% of patients with SHAPS scores indicating "no anhedonia" at baseline, 42.86% of patients with "mild/moderate" anhedonia, and 45.28% of patients with "severe" anhedonia were clinical responders (*χ* ^2^ = 0.43, *p* = .81). Similar results were seen for remission versus non‐remission: 47.06% of non‐anhedonic patients at baseline, 33.33% of mildly/moderately anhedonic patients, and 29.25% of severely anhedonic patients were IDS‐SR remitters (*χ* ^2 ^= 2.16, *p* = .34).

Additionally, a binary logistic regression model with baseline SHAPS, gender, and age as independent variables and clinical outcome as the dependent variable did not reveal baseline anhedonia to be a significant predictor for either IDS‐SR response or remission outcomes. For response versus nonresponse as the dependent variable, baseline SHAPS score showed a *χ* ^2^ of 0.77 (*p* = .38, *df* = 1), and the overall model showed *χ* ^2 ^= 0.93 (*p* = .82) with a 54.2% successful prediction of clinical outcome with a Nagelkerke *R* ^2^ of 0.01, indicating that the model with baseline SHAPS score as an independent variable did not predict response versus nonresponse to TMS, and explained only 1% of the variability. Similar results for remission versus non‐remission were also observed (baseline SHAPS score *χ* ^2 ^= 2.26, *p* = .13, *df* = 1; overall model χ^2^ = 2.50, *p* = .48; 68.1% successful prediction of clinical outcome, and Nagelkerke *R* ^2^ = 0.02).

## 4 DISCUSSION

This is the first large, naturalistic treatment study to examine the effect of standard, non‐research TMS on the symptom of anhedonia and evaluate whether patients with severe anhedonia are good candidates for TMS therapy. A specific assessment tool for anhedonia was used and TMS treatment outcomes for depressed patients, where the outcome variables (response, remission) were calculated on an independent scale with and without including its hedonic drive items, were tested.

Our results indicate that among depressed patients, a course of TMS therapy resulted in a statistically and clinically significant improvement in anhedonia. The degree of improvement in anhedonia symptoms was also comparable to the degree of improvement seen in other symptoms of depression. Our data also showed that the severity of baseline anhedonia was not associated with the likelihood of reaching clinical response or remission after TMS. We found that the likelihood of response or remission following a standard course of TMS was not statistically worse (nor statistically better) for patients with severe baseline anhedonia than it was for depressed patients with mild/moderate anhedonia or no anhedonia. This finding persisted even when the TMS treatment 50% “response” outcome was calculated after removal of the four items on the IDS‐SR scale which were intended to assess aspects of anhedonia. Our finding that degree of improvement in overall depressive symptoms was not predicted by baseline anhedonia scores stands somewhat in contrast to previous studies which found that anhedonia prior to TMS treatment was associated with depressive symptom improvement following TMS (Downar et al., 2014), or that baseline anhedonia played a significant part in predicting clinical nonresponse (Rostami et al., 2017). However, both of those studies ultimately defined anhedonia by individual items taken from one or more general self‐report depression scales while searching for potential symptom clusters from a myriad of measures to correlate with clinical outcomes. In contrast, the methods in our study involve a specific anhedonia measure.

Rostami et al. (2017) found that the score selected by patients on the Beck Depression Inventory‐II (BDI) single item (#12) “loss of interest” had a statistically significant impact on post‐treatment clinical response/nonresponse status as determined by the entire BDI via logistic regression. Similarly, Krepel et al. (2020) used a naturalistic database of patients receiving a combination of TMS and psychotherapy to investigate whether items from the BDI can be used as clinical predictors of treatment outcomes. They found that the sum score of three items (#4: “dissatisfaction/loss of pleasure”; #12: “loss of interest”; and #21: “interest in sex/libido”) was negatively correlated with overall BDI percent change; however, it was not deemed clinically useful in predicting outcomes in a replication sample. Although the studies examined all individual items from the BDI, items found to be statistically significant predictors were heterogeneous even among the symptoms thought to belong in the anhedonic clusters. Notable differences between the studies of anhedonia and TMS include target site of stimulation, primary diagnosis for depressed patients, and combination of TMS with other concurrent interventions such as psychotherapy. A recent study by Siddiqi et al. (2020) has suggested that stimulation of the cingulo‐opercular network (also known as the ventral attention network) may be the optimal location for a depressive symptom cluster biotype that includes the anhedonic symptoms. Thus, further optimization of treatment target areas based on the symptom cluster of choice may be a clinical reality in the near future.

Limitations of this study include the fact that although SHAPS is used widely as a tool to study anhedonia, it is specific for the measurement of consummatory pleasure and does not cover other subconstructs of anhedonia such as motivation and interest/desire. In recent years, newer assessment tools have been constructed to test the other domains of anhedonia as well (Light et al., 2019; Rizvi et al., 2016). Studies have demonstrated that these subconstructs may have distinct neurochemical pathways and neuroanatomical regions associated with them, including the prefrontal cortex, striatum, amygdala, and the nucleus accumbens (Der‐Avakian & Markou, 2012; Siddiqi et al., 2020; Treadway & Zald, 2011). Therefore, examining the other aspects of anhedonia may be of interest in the future, as each of the aforementioned areas of anhedonia may respond differently to treatment or may be affected in a unique manner depending on depression subtypes. Additionally, the data are retrospective and derived from a naturalistically treated sample. While there was considerable variability in the use of concurrent medications and psychotherapies during the course of TMS, we are confident that our sample represented the type of patients encountered in regular clinical TMS settings, rendering the results relevant to standard clinical practice.

## 5 CONCLUSIONS

A standard course of TMS treatment for MDD is effective for improving anhedonia, specifically aspects related to loss of consummatory pleasure. While the severity of the deficit in experiencing consummatory pleasure prior to treatment did not predict clinical outcome following a standard course of TMS therapy, we found patients with severe baseline anhedonia had an equal chance of achieving clinical response or remission. Patients with marked anhedonia should not be excluded from TMS treatment if they are safe for outpatient care and otherwise appropriate candidates for magnetic stimulation.

### PEER REVIEW

The peer review history for this article is available at https://publons.com/publon/10.1002/brb3.2329

## Data Availability

The data that support the findings of this study are not publicly available, but are available from the corresponding author upon reasonable request.
